# Advanced P Wave Detection in Ecg Signals During Pathology: Evaluation in Different Arrhythmia Contexts

**DOI:** 10.1038/s41598-019-55323-3

**Published:** 2019-12-13

**Authors:** Lucie Maršánová, Andrea Němcová, Radovan Smíšek, Martin Vítek, Lukáš Smital

**Affiliations:** 1 Department of Biomedical Engineering, Faculty of Electrical Engineering and Communication, Brno University of Technology Technická 12, Brno, 616 00 Czech Republic; 2 Institute of Scientific Instruments, The Czech Academy of Sciences Královopolská 147, Brno, 612 64 Czech Republic

**Keywords:** Cardiac device therapy, Biomedical engineering

## Abstract

Reliable P wave detection is necessary for accurate and automatic electrocardiogram (ECG) analysis. Currently, methods for P wave detection in physiological conditions are well-described and efficient. However, methods for P wave detection during pathology are not generally found in the literature, or their performance is insufficient. This work introduces a novel method, based on a phasor transform, as well as innovative rules that improve P wave detection during pathology. These rules are based on the extraction of a heartbeats’ morphological features and knowledge of heart manifestation during both physiological and pathological conditions. To properly evaluate the performance of the proposed algorithm in pathological conditions, a standard database with a sufficient number of reference P wave positions is needed. However, such a database did not exist. Thus, ECG experts annotated 12 chosen pathological records from the MIT-BIH Arrhythmia Database. These annotations are publicly available via Physionet. The algorithm performance was also validated using physiological records from the MIT-BIH Arrhythmia and QT databases. The results for physiological signals were Se = 98.42% and PP = 99.98%, which is comparable to other methods. For pathological signals, the proposed method reached Se = 96.40% and PP = 85.84%, which greatly outperforms other methods. This improvement represents a huge step towards fully automated analysis systems being respected by ECG experts. These systems are necessary, particularly in the area of long-term monitoring.

## Introduction

Cardiovascular diseases are currently the most common cause of death worldwide^1^. Due to the simplicity of the method, its noninvasive character, and the cost effective nature of the procedure, electrocardiography is still the most available and widely used method for the examination of the heart’s electrical activity^2^. An electrocardiogram (ECG) reflects the electrical activity of the heart and provides a significant amount of information about heart function, which is required for the correct diagnoses of various diseases. An automatic analysis of an ECG is a fundamental task in cardiac monitoring, particularly in the case of long-term monitoring, where large amounts of data are recorded^3^. Manual evaluation of such data is extremely time-consuming. Therefore, there is a significant effort to improve conventional methods and to develop new, accurate, and robust methods for processing and analyzing electrocardiographic records.

Automatic detection of the QRS complex, P wave, and T wave, represents one of the most important steps towards identification of pathology in an ECG^4^. The correct detection of these ECG components is crucial for accurate diagnoses. Detection of the P wave is the most complicated part of the process, and it remains challenging. The reasons for this are: (a) P waves have a low voltage, resulting in a low signal-to-noise ratio (SNR); (b) P waves have no exclusive time and frequency characteristics; (c) P waves have high interpatient variability; (d) in the case of atrioventricular (AV) dissociations, P waves do not respect normal time ordering of an ECG sequence (P wave can be missing, redundant, or even hidden within the QRS complex or T wave); (e) in the case of tachycardia, P waves can be hidden within T waves^3^.

Currently, a large range of commercial software solutions are used for automatic analysis of long-term ECG^5–9^ in practice; however, none of them can reliably evaluate ECG records. It is still necessary to have records reviewed by a trained ECG expert or a cardiologist^10^. These software solutions generally offer the following features: detection of ventricular and supraventricular premature beats (also called ‘premature ventricular contraction’ (PVC)), evaluation of heart rate variability in the time and frequency domain, pacemaker analysis, analysis of atrial fibrillation in the time and frequency domains, analysis of the ST segment, measurement of the QT interval, detection of R wave within T wave, and detection of first-degree AV block^5–9^. However, none of these can automatically detect pathologies as the identification is based on a correct detection of P waves, which do not respect normal time ordering. The correct detection of P waves in these situations is necessary for diagnosis of pathologies, such as second- and third-degree AV block, as well as some types of supraventricular tachycardia with AV dissociations^2^.

This paper introduces a new method for P wave detection, which is able to detect P wave during second-degree AV block (AVB II), as well as signals containing ventricular extrasystoles. Second-degree AV block is a heart disease that arises when the conduction of atrial impulses through the AV node is blocked. This means that only the atrial contraction is present, but the contraction of ventricles is not. In ECG signals, not all P waves are followed by a QRS complex. Ventricular extrasystoles originate in the ventricles and occur earlier than normal sinus beats. These extrasystoles are generally associated with a wide QRS complex, a full compensatory pause, and an absence of the P wave^10^. Patients can be asymptomatic or can experience syncope or palpitations. Both pathologies are associated with an increased risk of mortality^11^.

For both of the previously described pathologies, it is typical that the ECG includes P waves that do not respect normal time ordering of the ECG sequence P-QRS (in the second-degree AV block, some P waves are not followed by a QRS complex, and in ventricular extrasystoles, P waves are missing)^2^. This is a significant problem for commonly used algorithms, because they generally rely on the relationship between the P wave and the QRS complex (the P wave is followed by the QRS complex), which is the case for a normal cardiac rhythm. The first step in these common methods is to search for the P wave in a localized area outside (before) the QRS-T. This area is demarcated with respect to RR interval and QRS complex fiducial points (onset and offset). Once the area has been defined, the maximum of P wave is generally detected directly, e.g., by adaptive threshold estimation^12,13^, using wavelet transform (WT)^14–16^, through extraction of P wave template and the application of correlation^17^, Kalman filtering^18^, moving average^13^, support vector machine^19,20^, Prony’s method^21^, the hidden Markov models^22^, a neural network^23^, phasor transform (PT)^24,25^, dynamic programing^26^, or combination of several detection algorithms^27^. These approaches deliver good results only in the cases of ECGs with normal cardiac rhythm, but these approaches were not tested on pathological records.

Only a small number of algorithms were tested on signals with pathologies, namely, second-degree AV block, nodal rhythm (NOD), and various types of ventricular abnormalities^3,4^. However, they do not provide good results (e.g., for PVC, the algorithm^3^ achieved sensitivity (Se) = 70.37% and positive predictivity (PP) = 59.41%, and the algorithm^4^ achieved Se = 76.14% and PP = 55.87%; in the case of AV block, the algorithm^3^ achieved Se = 72.79% and PP = 99.51%, while the algorithm^4^ achieved Se = 49.76% and PP = 99.76%).

For testing an algorithm, physiological as well as pathological ECG records are needed. The P wave positions were manually annotated by experts. None of the existing publicly available ECG databases met these requirements. QT database (QTDB), is annotated in terms of P wave;^28,29^ however, QTDB contains only physiological records and the annotations are not publicly available. Elgendi et al^30^. published annotations of the whole MIT-BIH Arrhythmia Database (MITDB). The ECG experts^31^ carefully checked the annotations and found out that they contain many mistakes. Thus, the annotations are neither used in this study as a reference nor can be recommended to other authors (e.g. some P waves are not marked although they are present, some P waves are marked although they are not present and some parts of signals have no annotations). Therefore, the ECG experts^31^ manually annotated 12 signals from the MITDB^32,33^. These annotations had been published previously on Physionet^34^.

## Methods

**Figure 1:**
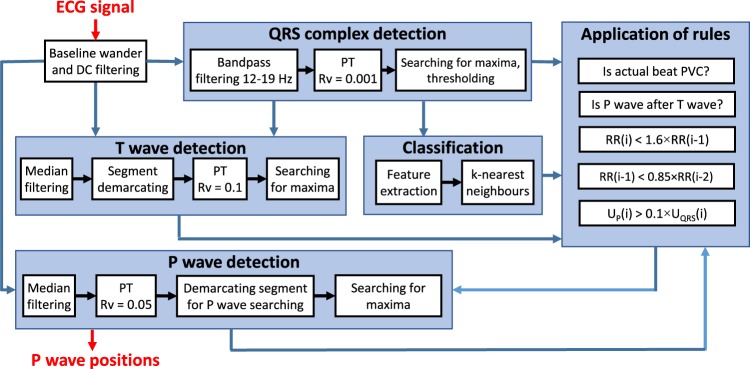
The overall process of P wave detection (filtration of ECG signal, QRS complex detection, T wave detection, classification of heartbeats (PVC/other), application of decision rules (decision whether the P wave can occur or not), and finally, P wave detection).

As the basis for P wave detection, the phasor transform^24^ is used. Additionally, detection of PVC and several novel decision rules are used to even more accurately detect a P wave, primarily in pathological cases. These rules are based on knowledge of heart manifestations during both physiological and pathological heart functions. Morphological features are derived from each QRS complex for use during detection of PVC (e.g., amplitude, width, area under the curve, and differences between actual and previous QRS complexes). The entire algorithm consists of eight parts: filtration, QRS complex detection, T wave detection, feature extraction, classification, application of decision rules (decision whether the P wave can be expected and what particular type it is, i.e., normal or dissociated), demarcation of a segment for P wave searching, and P wave detection. The whole process is demonstrated in Fig. 1. Each of the blocks are thoroughly described in the sections that follow.

### Phasor transform

In 2010, Martinéz *et al*. published an original paper on the automatic delineation of ECG fiducial points (P wave onset, peak and offset, QRS onset and offset, and T wave peak and offset) using phasor transform^24^. PT enhances variations of the signals (in this case, P wave, T wave, and QRS complex), thereby making the detection of these components easier. The PT transforms each sample of the original ECG signal, *x*(*n*), into a phasor signal, *y*(*n*), where *n* is discrete time. The real part of the signal is a constant value *R*_*V*_, which is set by the user, and the imaginary part of the phasor signal is the original value of the actual sample *x*(*n*). The phasor *y*(*n*)can be defined for each sample as follows:

1 $$y(n)={R}_{V}+jx(n).$$

From the phasor, magnitude *M*(*n*) and phase *φ*(*n*) are calculated according to equations (2) and (3). These two signals can be computed as follows:

2 $$M(n)=\sqrt{{R}_{V}^{2}+x{(n)}^{2}}$$

3 $$\varphi (n)=ta{n}^{-1}(\frac{x(n)}{{R}_{V}})$$

Phase *φ*(*n*) is mainly used for P wave detection. This signal is normalized to the interval <0, π/2>. Magnitude *M*(*n*) is used as one additional criteria. The efficiency of the enhancement of the ECG components can be controlled by setting *R*_*V*_. The value of *R*_*V*_ is always within the interval <0, 1>. If the *R*_*V*_ is low, phase differences are higher, and thus, changes in signal are highlighted. The values of *R*_*V*_ were first set according to a previous study^24^, then they were heuristically optimized.

**Figure 2:**
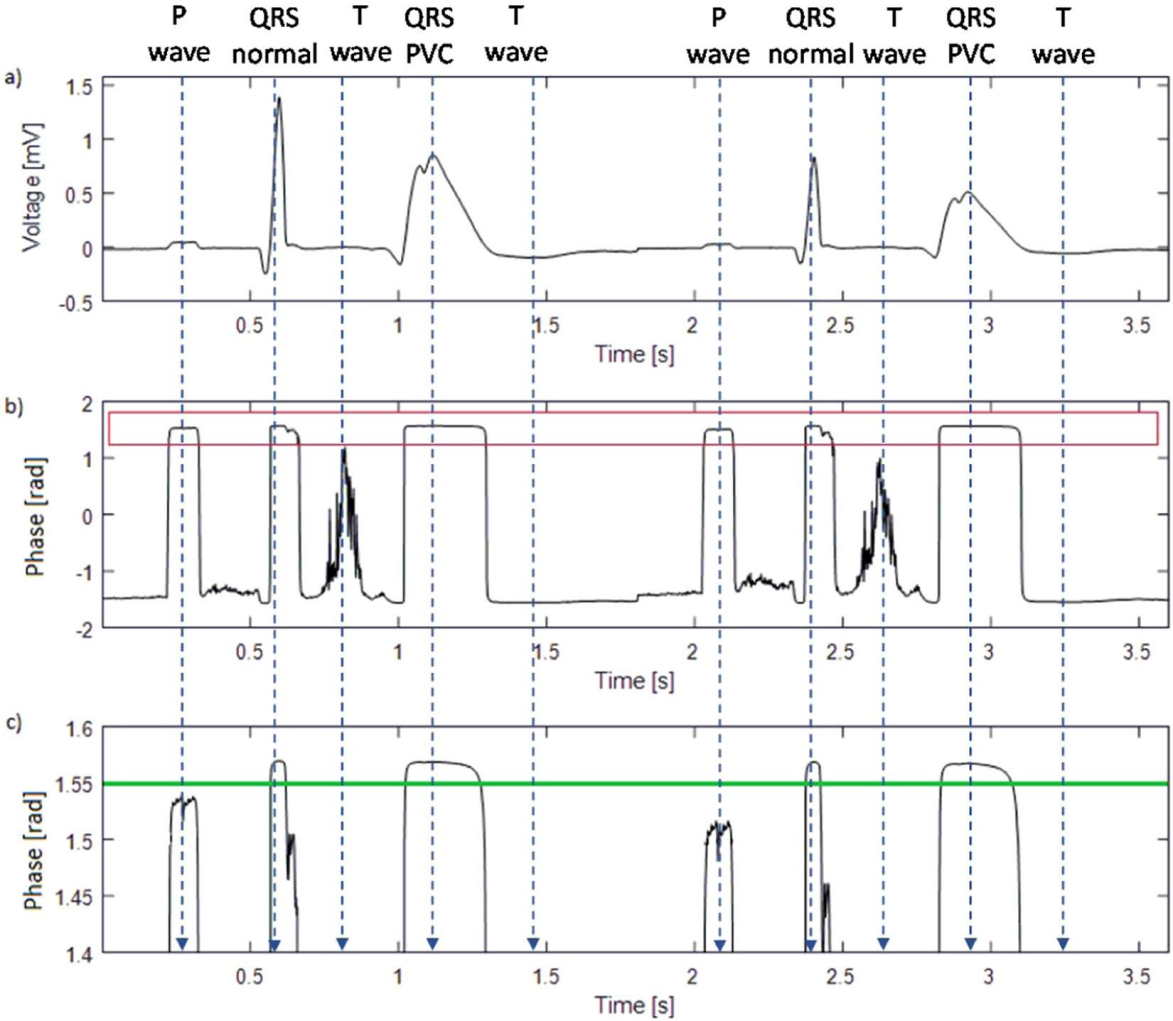
An example of (**a**) an original ECG signal; (**b**) phase signal *φ*(*n*); (**c**) detail of the top part of the phase signal (illustrated by red box in (**b**)), the green line represents the threshold for QRS detection.

Figure 2 shows an example of a phase signal (b) obtained by the phasor transform of the original ECG signal (a). Figure 2(c) indicates in detail the area of interest for the phase signal, which is highlighted by a red rectangle in Fig. 2(b). In the phase signal, *φ*(*n*), all ECG components are notably enhanced and thus easier to detect than in the original signal. The QRS complexes have the highest amplitude of all of the ECG components, even when the original ECG signal has a smaller amplitude of QRS complexes than T waves. This is because the altitude in the phase signal increases alongside an increasing frequency (QRS complex includes higher frequency components than T wave). However, P waves and T waves are significantly enhanced and are easily detectable in the phase signal.\

### Testing database and newly published P wave annotations

For testing a proposed algorithm, physiological as well as pathological ECG records, with P waves annotated by experts, were needed. However, records with pathology and manual annotations (provided by a human expert) are not publicly available or contain mistakes^30^. Therefore, ECG experts manually annotated 12 signals from the MITDB. These annotations were published on Physionet^29,34^, where they are freely available. This dataset represents roughly six hours of ECG. Signals identical to those chosen by the authors in^3^ were selected in order to enable a comparison. The authors of^3^ also manually annotated part of these signals but the annotations are not publicly available; further, they did not specify which parts of the signals were annotated. For the sake of completeness, there is also the QTDB, which is annotated in terms of P wave^28,29^. However, only between 30 and 100 normal beats in each signal are annotated.

### MIT-BIH Arrhythmia Database

The MIT-BIH Arrhythmia Database^32,33^ is the most commonly used database for evaluation of QRS detectors, and it is the most cited database overall^35^. The MITDB includes reference positions for QRS complexes. The database consists of 48 half-hour two-channel ECG records. The sampling frequency is 360 Hz. We used the first lead of each ECG record. In most records, the first lead is a modified limb lead II (MLII), obtained by placing the electrodes on the chest.

From this database, 12 physiological and pathological signals were selected, specifically, signals no. 100, 101, 103, 106, 117, 119, 122, 207, 214, 222, 223, and 231. Records no. 106, 119, 207, 214, 222, 223 and 231 were selected because they include a large number of different pathologies. Records no. 106, 119, 214, and 223 include PVCs (various types of ventricular arrhythmias - ventricular bigeminy (B), ventricular trigeminy (T), and idioventricular rhythm (IVR)). Records no. 214 and 222 include nodal rhythm (NOD) and 231 includes second degree AV block. Records no. 100, 101, 103, 117, and 122 do not include any pathologies (or only a few). Therefore, these signals are considered to represent physiological signals, and they were used to verify the performance of the algorithm in physiological conditions.

Two experts^31,34^ performed the annotations of the 12 abovementioned signals: the first expert provided manual annotations, and the second checked them. Unclear parts of the records were checked by both experts until a consensus was reached. Everything was conducted manually without the use of automated software. To facilitate the work of ECG experts, the free software tool, SignalPlant^36^, was used for manual marking of P waves.

The experts annotated only the P waves, which are clearly visible by eye and not hidden in the T waves or in the QRS complexes. The annotations are meant to be used as a reference for the testing of software designed to detect visible P waves. The saved position corresponds to the peaks of P waves (positive or negative P waves), or the middle of P waves (biphasic P waves).

### QT database

The QT database^28,29^ is the most commonly used database for the evaluation of ECG delineation algorithms. The database includes 105 15-minute two-channel ECG records. The sampling frequency is 250 Hz. We used the first lead of each ECG record.

For all records and all beats, the automatically found reference positions of QRS complexes are available. For some beats, the QTDB also includes manual annotations of P wave peak, P wave onset, P wave offset, QRS complex onset, and QRS complex offset, as well as the position of the T wave peak and the T wave offset. All of these annotations are available for at least 30 beats per record in 79 out of the 105 recordings^28^.

The performance of the algorithm for the P wave and the T wave detection was tested against the manually annotated part of the QTDB (which includes 3,622 beats).

### Preprocessing

For the detection of each important ECG component (QRS complex, P wave, and T wave), the original signal was first preprocessed. The preprocessing aspect concerns filtering redundant components out of the original ECG signal, and enhancement of the important components. Baseline wander and low frequency components were eliminated using a high-pass Lynn’s filter with a recommended cut-off frequency of 0.67 Hz^37,38^. For the detection of the QRS complex, a bandpass FIR filter with Hamming window with cut-off frequencies of 12 Hz and 19 Hz^39^, was used for suppressing P waves and T waves. For detection of P waves and T waves, the ECG signal was smoothed by a median filter using a 40 ms sliding window.

### QRS complex detection

To detect QRS complexes, the PT with *R*_*V*_* = *0.001 was applied to the preprocessed ECG signals. Then, the maxima (potential QRS complexes) was searched for in phase signal *φ*(*n*) using a sliding window with a length of 300 ms. This value was derived based on physiological limits of heart rate. The sliding window of 300 ms in length shifted the phase signal, and the maximum was searched for in each window. The beginning of the window corresponded to the position of the previously discovered maximum. Following this, two decision rules were applied to the discovered points to exactly specify the detection of the QRS complexes. The first rule checked whether the discovered maximum was higher than the adaptive threshold. The threshold was calculated (determined) by doubling the standard deflection of the phase signal in a sliding window with a length of 2 s. This value was established heuristically. If the maximum was higher than the adaptive threshold, then this maximum was marked as the QRS complex; if not, it was discarded. The second rule checked whether all of the QRS complexes had been detected. The length of two subsequent RR intervals – current RR interval *RR*(*i*) and previous RR interval *RR*(*i*-1) – were compared. If *RR*(*i*) ≥ 1.75 × *RR*(*i*-1), backward searching of the QRS complex was performed, because one QRS complex was likely missing within the *RR*(*i*) segment. In this case, a search was made for the maximum within the *RR*(*i*) segment in the magnitude signal *M*(*n*). The threshold was set at 0.3 × the altitude of the previously detected R wave.

### Detection of premature ventricular contraction

Detection of premature ventricular contraction is a very important step for demarcating an area where P waves can occur. The detection comprises two steps, feature extraction and heartbeat classification. The results of classification are used to decide whether the P wave should be searched for. The P wave is not searched for if the actual QRS complex is classified as PVC, because the P wave is not present.

The basic version of the classification algorithm was presented in previously published work^34^. However, the result presented in that manuscript was only good for signals with PVC; the algorithm was not able to deal with signals that included other pathologies.

### Feature extraction

For classification purposes, six different morphological features were extracted from each QRS complex. These features are: a) the area under the curve (AUC) – calculated in the segment of the original ECG demarcated from *R*(*i*)−50 ms to *R*(*i*)+50 ms, where *R*(*i*) is the position of the current R wave; b) the difference between AUC of the actual and the previous QRS complex; c) the length of *RR*(*i*); d) the length of *RR*(*i-*1); d) the difference between *RR*(*i*) and *RR*(*i-*1); e) the altitude of the actual QRS complex *U*_*QRS*_(*i*); f) the difference between the altitude of the actual and the previous QRS complex, *U*_*QRS*_(*i*)−*U*_*QRS*_(*i-*1). Some of these features were previously published in studies^34,40^, where their usability was proven. All of these features can be computed in a simple manner, and they indicate significant differences between the normal heartbeats and PVC, as shown by the results of a nonparametric Kruskal-Wallis test (α = 0.05), followed by a Tukey-Kramer post hoc test^40^. These features were further used as input in the classification algorithm, to distinguish between PVC and other beats.

### Classification of heartbeats

For differentiation between PVC and other beats, the k-nearest neighbors (k-NN) method was used^40^. The k-NN algorithm is one of the simplest, and most-used machine learning classification algorithms. The number of neighbors was selected as one (k = 1). The classification algorithm was trained using the entire MITDB, with the exception of the 12 records on which the testing was performed (signals no. 100, 101, 103, 106, 117, 119, 122, 207, 214, 222, 223, and 231). The results of the classification were used for deciding whether the P wave was searched for before the actual QRS complex or not. If the beat is considered to be a PVC, the P wave is not present, and thus, the P wave is not searched for in this beat. Accuracy for the classification of type of beat for the 12 signals was 94.35%, sensitivity was 91.2% and specificity was 93.6%.

### T wave detection

For T wave detection, the searched area was determined using the positions of the QRS complexes. After each QRS complex *R*(*i*), the area was demarcated from *R*(*i*) + 0.16 × *RR*(*i* + *1*) to *R*(*i*) + 0.57 × *RR*(*i* + *1*). In this area, the ECG signal was transformed using the PT with *R*_*V*_ = 0.1. The maximum of phase signal *φ*(*n*) within the demarcated segment was considered as the position of the T wave. Information about the T wave position was subsequently used for demarcating the area for P wave detection.

### P wave detection

**Figure 3:**
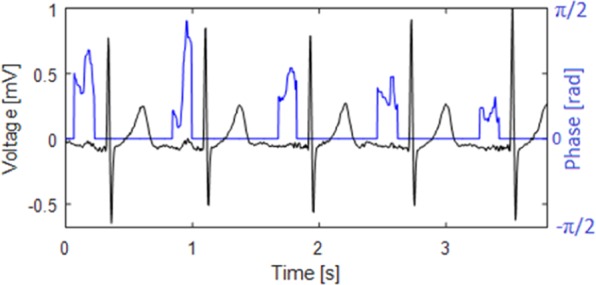
The example of the phase signal (gray color) obtained from the original ECG signal (black color) after PT application. Phase signal is ready for P wave detection. The third, fourth, and the fifth P wave, which are not clearly visible in the original ECG signal, are notably enhanced and easily detectable in the phase signal. PT is provided only for the segments demarcated according to QRS complex positions.

For P wave detection, it is necessary to know the positions of the QRS complexes and the T waves. According to the position of the QRS complex *R*(*i*), the algorithm demarcates the segment from *R*(*i*)−0.7 × *RR*(*i*) to *R*(*i*)−0.07 × *RR*(*i*). In this segment, the PT with *R*_*V*_ = 0.05 was applied to the filtered signal (see section 2.2). In the demarcated P wave segment of phase signal *φ*(*n*), the maximum was searched for, and this was considered to be a potential P wave. An example of an original ECG signal and the phase signal for the detection of the P wave is shown in Fig. 3. It is clear that P waves (which are not clearly visible in the original signal, particularly in the third, fourth, and fifth heartbeat) in the phase signal are significantly enhanced and therefore, easily detectable.

The detection of P waves according to the positions of the QRS complexes was established, precisely, using several novel additional criteria (decision rules). These innovative rules are based on knowledge of heart manifestations during both physiological and pathological heart functions. The first attempt was to use a basic version of criteria (c) and (e) in the previously published method for P wave detection during second-degree AV block^25^. These rules were improved, optimized, and combined with a classification algorithm. New rules, (a), (b), and (d) represent the most important innovations of the methods presented in this paper.

- **First**, according to the classification results, the algorithm can define whether the actual beat is normal or PVC. If the actual heartbeat is PVC, the position of the detected P wave is cancelled because the P wave is not present before PVC. If this situation occurs, the other criteria, (b), (c), (d), and (e) are skipped.
- **Second**, it is verified whether the detected P wave of an actual heartbeat is located after the T wave of the previous heartbeat. If this criterion is not met, it means that the T wave of the previous heartbeat was detected instead of the P wave. This means that the P wave is not present in the actual heartbeat, or it is hidden in the previous QRS complex or T wave. The position of the currently detected P wave (in fact, the T wave) is then deleted. If this condition is not met, criteria (c), (d), and (e) are skipped.
- The **third** criterion checks whether *RR*(*i*) > 1.6 × *RR*(*i-*1). If this criterion is met, it means that the dissociated P wave can be present in the beat due to the presence of a second-degree AV block. If the criterion is met and the previous QRS complex is not PVC, the dissociated P wave is searched for. To localize the dissociated P wave position, which is the PT, the same settings are applied as if searching for the normal P wave (before the QRS complex). The settings are applied to the filtered ECG segment, which begins 200 ms after the previous T wave position and ends 300 ms before the position of the actual QRS complex. The maximum detected in this segment is considered to be a potential dissociated P wave.
- If the third criterion is met, the result may have occurred due to the presence of a supraventricular extrasystole. Therefore, the **fourth** condition checks whether *RR*(*i-*1) < 0.85 × *RR*(*i-*2). If this condition is met, the algorithm assumes that the *RR*(*i*) is prolonged due to the presence of a supraventricular extrasystole, which is the beat *R*(*i-*1). This condition ensures no searching is done for the dissociated P wave because it is not present in the actual prolonged RR interval.
- The **fifth** criterion examines the voltage of the detected P wave, *U*_*P*_(*i*) > 0.1 × *U*_*QRS*_(*i*). If this criterion is met, the potential P wave is now considered to be a real P wave. If it is not met, the position of such a P wave is deleted. This is applied for all types of P waves.

To unify the marking of P wave positions, the peak of the P wave was chosen and searched as the maximum in the original ECG signal segment, which begins 20 ms before and ends 20 ms after the maximum detected in the phase signal *φ*(*n*). All numerical values were determined according to heart physiology and subsequently empirically adjusted.

**Figure 4:**
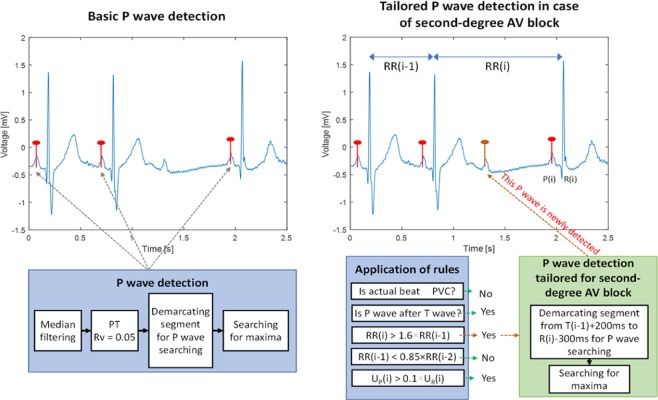
An example of the P wave detection procedure and the application of decision rules in the case of ECG signal with second-degree AV block. In the first stage (left image), three P waves (before QRS complexes) are found. In the second stage, the fourth P wave is detected as a result of the decision rules. Specifically, (a) *R*(*i*) is not PVC; (b) P wave follows T wave; (c) *RR*(*i*) is longer than 1.6 × *RR*(*i-*1). This means that additional searching for the P wave within the area from *T*(*i*)-200 ms to *R*(*i*)-300 ms is provided and a new P wave is found (the QRS complex following this beat was blocked due to the second-degree AV block; (d) *RR*(*i*-1) is not shorter than 0.85 × *RR*(*i*-2); (e) *U*_*P*_(*i*) is higher than 0.1 × *U*_*R*_(*i*).

**Figure 5:**
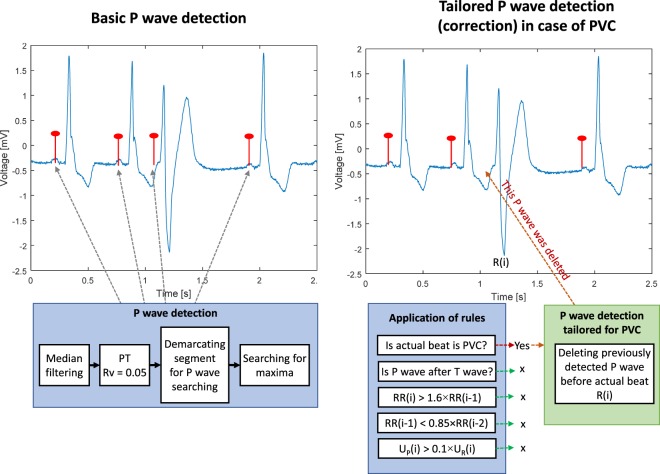
An example of the P wave detection procedure and the application of decision rules in the case of an ECG signal with PVC. In the first stage (left image), four P waves (before QRS complexes) are found. In the second stage, the third P wave is deleted due to the first decision rule; the third beat (*R*(*i*)) is PVC, which means that the P wave detected during the basic P wave detection *R*(*i*) is deleted. The remaining criteria (marked with x) were not checked further.

Figures 4 and 5 present examples of P wave detection during pathology, using the afore- mentioned criteria. In Fig. 4, detection of P waves is shown in the signal with second-degree AV block. Figure 5 shows the detection of P waves in a signal with PVC. Details are described in the title of each Fig.

## Results and Discussion

The most important part of this work is the significant progress presented in the area of P wave detection in pathological records. To support this statement, pathological signals no. 106, 119, 207, 214, 222, 223, and 231 from the MITDB were used. To validate that the algorithm was accurate in signals with a physiological rhythm, records no. 100, 101, 103, 117, and 122 were used. The algorithm was able to detect all possible types of P waves (positive, negative and biphasic).

For comparison, formerly published algorithms^16,25^, were tested on the same physiological and also pathological signals from MITDB. The algorithm^25^ is a previous version of the proposed algorithm, in which only PT and searching for P wave in demarcated area before QRS complex are used. The method described in^16^ utilizes continuous WT (wavelet bior1.5, scale 41) and searching for P wave in the area between T wave of previous beat and onset of QRS complex.

For a more thorough comparison, proposed algorithm as well as various well-known methods^15,18,24,26^ were tested on the manually annotated part of the QTDB. The methods^15^ and^24^ are based on dyadic WT (wavelet quadratic spline) and PT, respectively. In both methods, the P wave is searched for in demarcated area according to the QRS complex position. The method^18^ uses nonlinear dynamic model and an extended Kalman filter for estimating the parameters of the model. Authors of^26^ solve the problem of P wave detection by using two mixture Gaussian function and the dynamic programming.

In addition, the algorithm was compared to algorithms of other authors, which were tested during pathological conditions, i.e., ecgpuwave^4^ and PP rhythm tracking^3^. The ecgpuwave method^4^ is based on digital analyses of the slope, amplitude, and width of the signal in a moving integrating window and searching for P waves before QRS complexes. The method^3^ is based on the knowledge of cardiac physiology and pathophysiology. The method relies on a QRS detection coupled with a P wave’s rhythm estimation to support P wave detection in the case of AV dissociations. It consist of 5 stages: filtering, QRS detection, P wave occurrence estimation, area selection, and finally P wave detection.

Testing of the algorithm^4^ was conducted by the authors of^3^. However, these algorithms were tested only on the parts of the signals that were used by us. The authors of^3^ did not clearly specify which parts of the signals they used. The only available information in the manuscript is that it comprised roughly five hours of physiological rhythm and 51 minutes of records with pathological conditions.

### Detection of P waves in physiological conditions

The proposed algorithm was tested on records no. 100, 101, 103, 117, and 122, taken from the MITDB, in order to validate that the algorithm is also efficient in signals without any pathology (with physiological rhythm). The results are summarized in Table 1. The results of the newly proposed method are considerably better than the results mentioned in previous works^16,25^, and comparable with algorithms^3,4^. The worse results of algorithms^16,25^ on MITDB are probably due to the fact that the algorithms are designed for short-term signals on which they have also been tested.

**Table 1:** The performance of P wave detection algorithms on physiological signals from MITDB (Se – sensitivity; PP – positive predictivity; Nr – not reported; PT – phasor transform; WT – wavelet transform).

Rec. No.	ecgpuwave^4^		PP rhythm tracking^3^		PT^25^		WT^16^		Proposed method	
	Se [%]	PP [%]	Se [%]	PP [%]	Se [%]	PP [%]	Se [%]	PP [%]	Se [%]	PP [%]
100	Nr	Nr	Nr	Nr	100.0	99.30	99.34	99.91	99.69	99.25
101	Nr	Nr	Nr	Nr	99.84	99.79	98.02	99.95	98.93	99.39
103	Nr	Nr	Nr	Nr	46.76	41.84	39.95	40.53	98.80	100.0
117	Nr	Nr	Nr	Nr	100.0	99.93	99.48	100.0	96.48	99.93
122	Nr	Nr	Nr	Nr	52.35	34.25	52.41	34.77	98.18	100.00
Mean	99.68	99.84	99.57	99.83	79.79	75.02	77.84	75.03	98.42	99.98

For a more thorough comparison of the proposed method with those of the other authors, the algorithm was tested on the manually annotated part of the QTDB. In this instance, there is absolute certainty that the other authors have used exactly the same data. The proposed method was compared with various well-known methods based on phasor transform^24^, wavelet transform^15^, and correlation analysis^17^. The results are summarized in Table 2. The outcomes achieved by the newly proposed algorithm are better in terms of sensitivity and positive predictivity compared to results presented in previous works. The previous works were not developed with respect to the pathological signals and thus, these algorithms fail in testing on QTDB in which the pathological beats are present occasionally.

**Table 2:** The performance of the P wave detection algorithms on physiological signals from the manually annotated part of QTDB (Se – sensitivity; PP – positive predictivity; PT – phasor transform; WT – wavelet transform).

Method	Se [%]	PP [%]
Proposed method	99.84	99.84
PT^24^	99.28	99.75
WT^15^	98.87	91.03
Correlation of template^17^	99.63	99.83
Mixture Gaussian^26^	96.13	97.70

### Detection of P waves in pathological conditions

The proposed algorithm was tested on records no. 106, 119, 207, 214, 222, 223, and 231, taken from the MITDB, which include several types of pathology. In Table 3, the results of testing are presented for each record separately. Table 3 also includes comparisons with previously published works^16,25^, which were tested using the same signals. In the second column, the type of pathology that prevails in the particular signal is noted. The results of the proposed method are notably better than the results in previously published works^16,25^. The results for each record individually are not available for algorithms^3,4^. Comparisons with these algorithms are provided in Table 4.

**Table 3:** The performance of P wave detection algorithms on signals with pathology, selected from the MITDB (Se – sensitivity; PP – positive predictivity; PT – phasor transform; WT – wavelet transform).

Rec. No.	Type of pathology	PT^25^		WT^16^		Proposed method	
		Se [%]	PP [%]	Se [%]	PP [%]	Se [%]	PP [%]
106	PVC	90.98	91.83	99.87	74.25	99.37	94.75
119	PVC	99.38	99.69	99.51	78.06	93.15	97.8
207	NOD	81.54	56.58	97.81	66.67	95.47	68.85
214	PVC	98.55	99.5	99.55	88.14	96.9	94.45
222	NOD	82.28	44.17	99.12	50.24	91.96	54.32
223	PVC	94.62	93.72	99.95	80.75	99.48	92.27
231	AVB II	78.39	99.68	78.03	99.05	98.5	98.45
Mean		89.39	83.59	96.26	76.74	96.40	85.84

**Table 4:** The performance of the P wave detection algorithms on manually annotated signals from MITDB; evaluation in different arrhythmia content (Se – sensitivity; PP – positive predictivity; PVC – premature ventricular contraction (includes all types of ventricular arrhythmias i.e., ventricular bigeminy (B), ventricular trigeminy (T), idioventricular rhythm (IVR)), NOD – nodal rhythm, AVB II – second-degree atrioventricular block; PT – phasor transform; WT – wavelet transform).

Pathology	ecgpuwave^4^		PP rhythm tracking^3^		PT^25^		WT^16^		Proposed method	
	Se [%]	PP [%]	Se [%]	PP [%]	Se [%]	PP [%]	Se [%]	PP [%]	Se [%]	PP [%]
PVC	76.14	55.87	70.37	59.41	99.72	80.30	95.88	96.18	97.23	94.82
NOD	87.50	21.60	42.50	18.09	90.91	79.35	98.47	58.45	93.72	61.59
AVB II	49.76	99.76	72.79	99.51	78.39	99.68	78.03	99.05	98.50	98.45

In Table 4, the results of P wave detection in signals with different arrhythmia content are shown. The results of the proposed method were compared with previously published works^16,25^ as well as with works of other authors, i.e.,^3,4^. Se and PP of the P wave detection in signals with PVC were computed as means of the results for records no. 106, 119, 214, and 223; in signals with NOD as means of the results for records no. 207 and 222; results for AVB II correspond with the results for record no. 231. These results are applicable to the proposed method and algorithms in^16,25^. The authors of^3,4^ also reported testing results for these types of arrhythmias. However, only parts of the signals were used (not the entire signal, as in this study), and the authors did not specify which parts. Thus, the comparison is only tentative. The results show that the algorithm is able to better detect P waves in all types of arrhythmia, mainly for PVC and AVB II, for which it was designed. The results for a nodal rhythm are slightly worse. Low PP for this pathology was obtained because during nodal rhythm, the P waves were not always present. At this time, the proposed algorithm cannot reveal this type of rhythm.

Based on the results, it is obvious that the initial classification of pathologies leads to significant improvement of P wave detection performance.

### Limitation of the study

The main limitation of this study is that the algorithm is focused only on a limited number of pathologies (PVC, AVB II). The algorithm is not able to properly detect P wave during e.g. atrial and ventricular fibrillation, flutter and junction rhythm. The main reason is an absence of a freely available standard ECG database with annotated P wave positions. For proper testing, it is necessary to annotate the signals in terms of P wave positions manually. This process is very time-consuming and needs ECG expert(s) involvement. Furthermore, the algorithm was not tested during presence of extensive noise and artifacts, therefore it cannot be guaranteed successful P wave detection in these situations.

## Conclusion

In this article, a highly efficient algorithm is proposed for P wave detection. The algorithm is based on phasor transform, and newly designed rules based on knowledge of heart manifestations and classification (detection of PVC). These decision rules and results of PVC detection enable finding the P wave in the correct location, or alternatively, to not search for it at all. For the purpose of testing pathological signals, there is no available standard ECG database with a sufficient number of correctly annotated P wave positions. Thus, ECG experts manually annotated 12 signals from the MITDB. These annotations are publicly available on Physionet^29,34^. The same signals were used in study^3^. The proposed algorithm can detect the P wave in both physiological ECG signals (tested on QTDB and signals no. 100, 101, 103, 117, and 122 from MITDB), as well as in signals with a pathology, namely, second-degree AV block and premature ventricular contraction (tested on signals no. 106, 119, 207, 214, 222, 223, and 231 from MITDB). For the physiological signals, the algorithm achieved a Se = 99.84% and a PP = 99.84% using the QTDB, and a Se = 98.42% and a PP = 99.98% using the MITDB. For pathological signals, the algorithm reached an overall Se = 96.40% and PP = 85.84% (each pathology separately: Se = 97.23% and PP = 94.82% for PVC; Se = 98.50% and PP = 98.45% for second-degree AV block; Se = 93.72% and PP = 61.59% for NOD). For the physiological signals, the achieved results are comparable with the results of other methods, and the results highly outperform the other methods for the pathological signals. This improvement represents a significant step towards fully automated analysis systems respected by ECG experts, which are highly needed, particularly in the area of long-term monitoring.

## References

[1] Mendis, S., Puska, P. & Norrving, B. Global atlas on cardiovascular disease prevention and control (2011).

[2] Kusumoto Fred M. (2009). ECG Interpretation: From Pathophysiology to Clinical Application.

[3] Portet F (2008). P wave detector with PP rhythm tracking: evaluation in different arrhythmia contexts. Physiological Measurement.

[4] Laguna P, Jané R, Caminal P (1994). Automatic Detection of Wave Boundaries in Multilead ECG Signals: Validation with the CSE Database. Computers and Biomedical Research.

[5] Cardio Day Holter ECG. GE HealthCare. https://www.gehealthcare.co.uk/en-gb/products/diagnostic-cardiology/ambulatory-ecg (2018).

[6] EKG Holter Cardio Track. SEIVA: Cardiology manufacture http://www.seiva.cz/products/holter-ekg/ (2018).

[7] Biomedical Systems Century C3000 Holter System Specifications. METEC: Marketing of speciality products for cardiology laboratories and hospital wards in Denmark and Sweden http://www.metec.dk/biomedsys/specs_C3000.html (2018).

[8] Cardio Visions Professional 24 hour Holter ECG Software for CardioMera. Meditech: 24-hour Ambulatory Blood Pressure Monitors & Holter ECG Devices http://www.meditech.hu/24-hour-holter-ecg-software-cardiomera.html (2018).

[9] Holter ECG. AMEDTEC – your partner in function diagnosis http://www.amedtec.de/downloads/Holter%20ECG.pdf (2018).

[10] Fisch C (2000). Centennial of the string galvanometer and the electrocardiogram. Journal of the American College of Cardiology.

[11] GOLDMAN, Mervin. Principles of Clinical Electrocardiography (Lange Medical Pubns, 1986).

[12] Elgendi, M., Jonkman, M. & De Boer, F. P wave demarcation in electrocardiogram. *2009 IEEE 35th Annual Northeast Bioengineering Conference* **1-2** (2009).

[13] Lin C (2014). Sequential beat-to-beat P and T wave delineation and waveform estimation in ECG signals: Block Gibbs sampler and marginalized particle filter. Signal Processing.

[14] Ghaffari A, Homaeinezhad MR, Akraminia M, Atarod M, Daevaeiha M (2009). A robust wavelet-based multi-lead electrocardiogram delineation algorithm. Medical Engineering & Physics.

[15] Martinez JP, Almeida R, Olmos S, Rocha AP, Laguna P (2004). A Wavelet-Based ECG Delineator: Evaluation on Standard Databases. IEEE Transactions on Biomedical Engineering.

[16] Vítek, M., Hrubeš, J. & Kozumplík, J. A Wavelet-Based ECG Delineation in Multilead ECG Signals: Evaluation on the CSE Database. *Proceedings of the World Congress on Medical Physics and Biomedical Engineering*, 177–80 (2009).

[17] Karimipour A, Reza AM (2014). Real-time electrocardiogram P-QRS-T detection - delineation algorithm based on quality - supported analysis of characteristic templates. Computers in Biology and Medicine.

[18] Akhbari M, Shamsollahi MB, Jutten Ch. ECG (2013). fiducial points extraction by extended Kalman filtering. Proceedings of the 36th International Conference on Telecommunications and Signal Processing.

[19] Mehta SS, Lingayat NS (2008). Development of SVM based classification techniques for the delineation of wave components in 12-lead electrocardiogram: A comparative evaluation. Biomedical Signal Processing and Control.

[20] Mehta SS, Lingayat NS (2009). Application of support vector machine for the detection of P- and T-waves in 12-lead electrocardiogram: A comparative evaluation. Computer Methods and Programs in Biomedicine.

[21] Niranjan UC, Murthy ISN (1993). ECG component delineation by Prony’s method: A comparative evaluation. Signal Processing.

[22] Graja S, Boucher JM (2005). Hidden Markov Tree Model Applied to ECG Delineation. IEEE Transactions on Instrumentation and Measurement.

[23] Carrault G, Cordier MO, Quiniou R, Wang F (2003). Temporal abstraction and inductive logic programming for arrhythmia recognition from electrocardiograms: A comparative evaluation. Artificial Intelligence in Medicine.

[24] Martínez A, Alcaraz R, Rieta JJ (2011). Application of the phasor transform for automatic delineation of single-lead ECG fiducial points. Physiological Measurement.

[25] Maršánová, L., Němcová, A. & Smíšek, R. Detection of P wave during Second-Degree Atrioventricular Block in ECG Signals. *Proceedings of the Student conference Blansko*, 55–58 (2016).

[26] Rao (2019). P- and T-wave delineation in ECG signals using parametric mixture Gaussian and dynamic programming. Biomedical Signal Processing and Control.

[27] Friganovic Kresimir, Kukolja Davor, Jovic Alan, Cifrek Mario, Krstacic Goran (2018). Optimizing the Detection of Characteristic Waves in ECG Based on Processing Methods Combinations. IEEE Access.

[28] Laguna P., Mark R. G., Goldberg A. & Moody G. B. A database for evaluation of algorithms for measurement of QT and other waveform intervals in the ECG. *Proceedings of the Computers in Cardiology*, 673–676 (1997)

[29] Goldberger AL (2000). PhysioBank, PhysioToolkit, and PhysioNet: Components of a New Research Resource for Complex Physiologic Signals. Circulation.

[30] Elgendi, M., Meo, M. & Abbott, D. A Proof-of-Concept Study: Simple and Effective Detection of P and T Waves in Arrhythmic ECG Signals. Bioengineering, **26** (2016). 10.3390/bioengineering3040026 PMC5597269 28952588

[31] Smíšek R (2016). M. CSE database: extended annotations and new recommendations for ECG software testing. Medical & Biological Engineering & Computing.

[32] MIT-BIH Arrhythmia Database P-Wave Annotations. Physionet https://physionet.org/physiobank/database/pwave/ (2018).

[33] Moody GB, Mark RG (2001). The impact of the MIT-BIH Arrhythmia. Database. IEEE Engineering in Medicine and Biology Society Membership.

[34] Maršánová, L. et al. Automatic Detection of P Wave in ECG During Ventricular Extrasystoles. *Proceedings of the World Congress on Medical Physics and Biomedical Engineering*, 381–85 (2018).

[35] Němcová A, Smíšek R, Maršánová L, Smital L, Vítek M (2018). A Comparative Analysis of Methods for Evaluation of ECG Signal Quality after Compression. Biomed Research International.

[36] Plesinger F, Jurco J, Halamek J, Jurak P (2016). SignalPlant: an open signal processing software platform. Physiological Measurement.

[37] Smital L, Vítek M, Kozumplík J, Provazník I (2013). Adaptive Wavelet Wiener Filtering of ECG Signals. IEEE Transactions on Biomedical Engineering.

[38] Kligfield P (2007). Recommendations for the standardization and interpretation of the electrocardiogram: part I: the electrocardiogram and its technology a scientific statement from the American Heart Association Electrocardiography and Arrhythmias Committee, Council on Clinical Cardiology. J. American College of Cardiology.

[39] Kohler BU, Hennig C, Orglmeister R (2002). The principles of software QRS detection. Engineering in Medicine and Biology Magazine.

[40] Maršánová L (2017). ECG features and methods for automatic classification of ventricular premature and ischemic heartbeats: A comprehensive experimental study. Scientific Reports.

